# Why digital medicine depends on interoperability

**DOI:** 10.1038/s41746-019-0158-1

**Published:** 2019-08-20

**Authors:** Moritz Lehne, Julian Sass, Andrea Essenwanger, Josef Schepers, Sylvia Thun

**Affiliations:** 1 Berlin Institute of Health (BIH), Berlin, Germany; 2 Charité – Universitätsmedizin Berlin, Berlin, Germany; 3 Hochschule Niederrhein – University of Applied Sciences, Krefeld, Germany

**Keywords:** Health policy, Health care

## Abstract

Digital data are anticipated to transform medicine. However, most of today’s medical data lack interoperability: hidden in isolated databases, incompatible systems and proprietary software, the data are difficult to exchange, analyze, and interpret. This slows down medical progress, as technologies that rely on these data – artificial intelligence, big data or mobile applications – cannot be used to their full potential. In this article, we argue that interoperability is a prerequisite for the digital innovations envisioned for future medicine. We focus on four areas where interoperable data and IT systems are particularly important: (1) artificial intelligence and big data; (2) medical communication; (3) research; and (4) international cooperation. We discuss how interoperability can facilitate digital transformation in these areas to improve the health and well-being of patients worldwide.

## Introduction

The digitalization of medicine promises great advances for global health. Electronic medical records, mobile health apps, medical imaging, low-cost gene sequencing as well as new sensors and wearable devices provide an ever-increasing flow of digital health data. Combined with artificial intelligence, cloud computing and big data analytics, this wealth of data holds huge potential for healthcare and can improve the lives of millions of patients worldwide – with better diagnostics, personalized treatments, and early disease prevention.^1–6^

But medical data are only useful if they can be turned into meaningful information. This requires high-quality datasets, seamless communication across IT systems and standard data formats that can be processed by humans and machines. Judged by these criteria, however, large parts of today’s medical data are virtually useless: Hidden in isolated data silos and incompatible systems, the data are difficult to exchange, process and interpret. In fact, the current medical landscape seems less characterized by “big data” but rather by a large number of disconnected small data. These are suboptimal conditions for the data-driven technologies anticipated to drive medical innovation. Uncovering the full potential of digital medicine requires an interconnected data infrastructure with fast, reliable and secure interfaces, international standards for data exchange as well as medical terminologies that define unambiguous vocabularies for the communication of medical information. In short: Digital health depends on interoperability.

The aim of this article is to show why interoperability is so important for achieving the full potential of digitalization in healthcare and medicine. Although the importance of interoperable health IT systems is increasingly acknowledged,^7–9^ awareness of this topic is still relatively low among healthcare professionals – especially compared with topics such as artificial intelligence, big data or mobile technologies, which are generally seen as the main drivers of digital health innovation.^6,10–13^ Accordingly, progress in health interoperability is slow.^14^ Here, we argue that interoperability is indispensable for advances in digital health and that it is, in fact, a prerequisite for most of the innovations envisioned for future medicine.

**Fig. 1:**
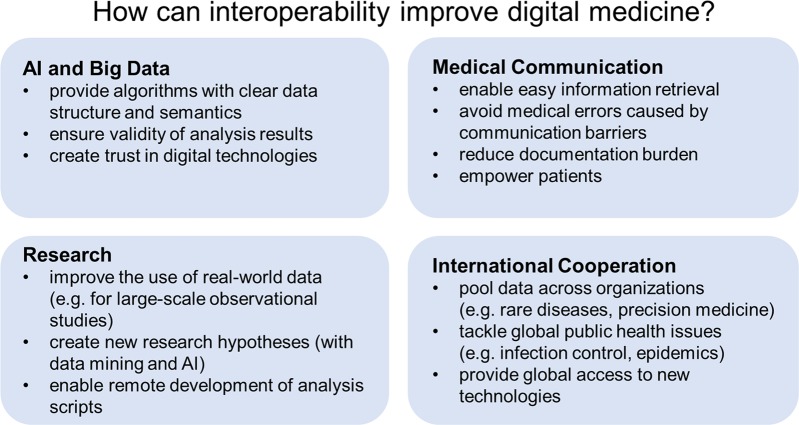
Overview showing how interoperability can improve medicine in the areas of artificial intelligence (AI) and big data, medical communication, research, and international cooperation

Our article starts with an overview of interoperability and its different levels: technical, syntactic, semantic, and organizational. It then shows how interoperability can improve medicine, focusing on four areas that especially benefit from (and sometimes crucially depend on) interoperable health IT systems: (1) artificial intelligence and big data; (2) medical communication; (3) research; and (4) international cooperation (Fig. 1). We chose these four areas because they illustrate particularly well how interoperability can facilitate digital transformation and improve medicine and healthcare (however, the areas are not mutually exclusive, and advancing, for example, medical communication can also improve international cooperation). Note that our views are shaped by our German/European perspective. However, we discuss points general enough to be relevant for international readers. Also note that, though giving some examples of specific health IT standards and medical terminologies that can improve interoperability, this article does not aim to provide detailed technical discussions of specific standards or terminologies (this information can be found elsewhere^15,16^).

## Interoperability

Interoperability can be broadly defined as “the ability of two or more systems or components to exchange information and to use the information that has been exchanged”.^17^ Most definitions further distinguish between different components, layers or levels of interoperability.^15,16^ Although these components can slightly differ across definitions, they generally follow a distinction between lower-level technical components and higher-level organizational components. In line with this conceptualization, this section gives a brief overview of technical, syntactic, semantic and organizational aspects of interoperability.

### Technical interoperability

Technical interoperability ensures basic data exchange capabilities between systems (for example, moving data from a USB stick to a computer). This requires communication channels and protocols for data transmission. With today’s digital networks and communication protocols, achieving technical interoperability is usually relatively straightforward. However, moving data from A to B is not enough. To process the data and extract meaningful information, syntactic and semantic interoperability is needed.

### Syntactic interoperability

Syntactic interoperability specifies the format and structure of the data (for example, in an XML document). The structured exchange of health data is supported by international standards development organizations (SDOs) such as Health Level Seven International (HL7) or Integrating the Healthcare Enterprise (IHE), which specify health IT standards and their use across systems. An emerging standard for the communication of health data is, for example, HL7’s Fast Healthcare Interoperability Resources (FHIR), which defines around 140 common healthcare concepts, so-called resources, which can be accessed and exchanged using modern web technologies.^18^ FHIR is increasingly adopted by the health industry, as it lends itself to the development of mobile health apps that run on different IT systems.^19^ Another initiative aiming to improve the structured exchange of health data is openEHR. OpenEHR allows medical professionals and health IT experts to define clinical content using so-called archetypes, specifications of clinical concepts based on an underlying reference model.^20^ OpenEHR includes a portable language for querying, the Archetype Querying Language (AQL), as well as tools for defining and publishing the archetypes.

### Semantic interoperability

While standards such as FHIR and openEHR already define basic semantics of health data, semantic interoperability is really the domain of medical terminologies, nomenclatures, and ontologies. They ensure that the meaning of medical concepts can be shared across systems, thus providing a digital “lingua franca”, a common language for medical terms that is, ideally, understandable to humans and machines worldwide. With more than 340,000 medical concepts (including, for example, clinical findings, procedures, substances, organisms, or body structures), the terminology SNOMED CT seems particularly well-suited as a general-purpose language for advancing semantic interoperability in medicine and healthcare.^21^ It can be complemented by more domain-specific terminologies such as, for example, Logical Observation Identifiers Names and Codes (LOINC) for laboratory observations,^22^ the Identification of Medicinal Products (IDMP) for medicines,^23^ the nomenclature of the HUGO Gene Nomenclature Committee (HGNC) for genes^24^ or the Human Phenotype Ontology (HPO) for phenotypic abnormalities.^25^ Combined with the standards discussed above, the use of these terminologies can ensure that health data have a clear structure and unambiguous semantics.

### Organizational interoperability

At the highest layer, interoperability also involves organizations, legislations and policies. Exchanging data across health IT systems is not an end in itself but should, ultimately, help healthcare professionals to work more efficiently and improve patients’ health. This requires common business processes and workflows that enable a seamless provision of healthcare across institutions. As different stakeholders in healthcare have different interests (and these interests do not always aim to maximize interoperability), this also requires policies that provide incentives for interoperable data exchange and, if necessary, enforce interoperability via legal regulations.

## How interoperability can improve medicine

### Interoperability for artificial intelligence and big data

Digital technologies such as artificial intelligence (AI) and the large-scale analytics subsumed under the term “big data” are increasingly changing medicine and healthcare.^6,10,12^ These technologies rely on growing volumes of digital medical data. Therefore, to use AI algorithms and big data analytics to their full capacity and feed them with maximum input, processing information from different systems and across institutional boundaries is crucial. A comprehensive analysis of a patient’s health data could, for example, require information from general practitioners, hospitals, laboratories, mobile health apps, and wearable sensors. Similarly, multiple data sources are often necessary when data are scarce, for example, in the areas of rare diseases, precision medicine, or pharmacogenomics: tailoring treatments and drugs to increasingly smaller subpopulations of patients requires a large pool of comparable data, making it necessary to exchange information across systems, institutions, and countries.

Unfortunately, today’s digital health infrastructure makes large-scale data processing across IT systems still unnecessarily difficult. Current health IT systems operate with a wide variety of data formats, custom specifications and ambiguous semantics. This situation is exacerbated by the trend to store increasing amounts of unstructured data in non-relational databases and so-called data lakes.^26^ Although these unstructured data are, arguably, better than no data – and modern algorithms can partly extract useful information even from unstructured data – they are difficult to process. As a consequence, time-consuming data cleaning and pre-processing procedures are usually necessary before analysis.

Moreover, running algorithms on unstructured, non-standardized data can introduce errors that distort analysis results. An AI algorithm programmed to identify, for example, diabetes patients from unstructured text could erroneously select patients with a family history of diabetes, not actual diabetes (not to mention the different types and subgroups of diabetes that could easily be confused). Such errors are difficult to detect in large datasets because the sheer volume of the data makes it difficult to anticipate, detect and correct all possible errors. This can introduce systematic biases, which compromise the validity of analysis results and which will eventually undermine trust in digital health technologies. This problem becomes even more relevant when considering the rise of artificial neural networks and deep learning algorithms. Although these algorithms can increasingly compete with (and even outperform) human experts,^27–29^ the mechanisms that drive their decisions usually remain hidden within the network. As these methods are essentially “black boxes” for human users, it is important that their calculations are based on a solid foundation. This requires data with a clear structure and unambiguous semantics. Otherwise, modern AI algorithms could do more harm than good – not because their calculations are wrong but because they rely on questionable input.

To avoid these pitfalls and provide AI algorithms and big data technologies with usable input, interoperability of health data is essential. The largest barrier for applying AI and big data to medicine is, arguably, not a lack of algorithms but a lack of suitable data for developing AI and big data applications. Using the international standards and terminologies mentioned previously can therefore help to provide algorithms with structured and meaningful data and foster the use of AI and big data in medicine.

### Interoperability for medical communication

Digital medicine does not always require sophisticated analytics or complex AI algorithms. In many cases, simply making the right information available to the right person at the right time can significantly improve patient care. Often, important parts of medical information are lost as patients move through the healthcare system. For example, if a patient is rehospitalized, relevant information from previous visits to other hospitals may not be available (in Germany, medical information can sometimes not even be shared across different departments of the same hospital due to data protection regulations). This leads to inefficiencies in care and sometimes poses serious risks for patients (for example, if a lack of communication results in adverse drug interactions). Giving healthcare providers the necessary information about their patients can help to avoid such inefficiencies and improve the quality of care.

Promoting the use of interoperable electronic health records (EHRs) is particularly important in this context. Too often, EHRs are disconnected, proprietary solutions buried in systems that do not talk to each other. The use of international standards and terminologies can make EHRs interoperable, enabling the reliable communication of medical information. Existing specifications for patient summary data such as the International Patient Summary (IPS) developed by HL7 and the European Committee for Standardization (CEN)^30^ can further help clinicians to access relevant, accurate, and up-to-date medical information about their patients.

Importantly, by making relevant health information easily accessible, interoperable health IT systems should also make lives easier for physicians and other healthcare professionals. The rise of digital health technologies often raises concerns that physicians have to spend more time with documentation and data entry and less time with their patients. But interoperable EHRs can reduce the documentation burden (for example, by avoiding repeated entry of data) and simplify cumbersome information retrieval processes. This can enable physicians to focus on their patients and provide optimal care.

On the other end of the spectrum, interoperable EHRs can also help patients to manage their own health more actively. Currently, much of the information that drives providers’ decisions about treatments is not easily accessible to the patients themselves, making them inactive bystanders in the treatment process. If patients are given better access to their health data (combining, for example, information about prescriptions, treatments and data from personal health apps), they can take more control of their health. This in itself can have positive health effects, as patients turn from helpless recipients of healthcare to active managers of their health and well-being.

### Interoperability for research

Apart from improving health at the point of care, interoperability can also advance medical research. This is particularly true for the field of real-world evidence: Using interoperable formats for real-world data (that is, data routinely collected in medical care or, increasingly, via mobile apps in patients’ everyday lives) opens up various opportunities for researchers. First, if real-world data are interoperable, they can be used for large-scale observational studies at regional, national or global levels. Such studies can address epidemiological questions and public health concerns, providing, for example, up-to-date insights into prevalence and incidence of disease, typical treatment pathways of patients or gaps in care. Second, real-world data are a treasure trove for the AI and machine learning methods discussed above. Being able to find patterns and correlations in high-dimensional datasets, these methods can help researchers to identify interesting new research hypotheses, which can subsequently be investigated more closely in controlled clinical trials (these controlled trials will remain important to rule out spurious correlations and identify causal relationships).

More generally, if health data are structured according to international standards, data are much easier to analyze, and efforts needed for data cleaning and pre-processing are reduced. This can speed up the research process and also makes the development of analysis scripts more flexible: If researchers and data scientists know that data will conform to certain formats and semantics, analyses no longer need to be programmed with direct access to the data. Instead, analyses can be developed remotely and later be transferred to the data site to compute results. This can unlock data sources that would otherwise not be easily accessible (due to, for example, strict data protection regulations). It can also improve research quality because analyses can be programmed by experts worldwide, not only by those who have direct access to the data (and who happen to know their idiosyncratic structure). Similarly, interoperable data can ensure that one analysis can be done across many different data sources, covering data from different institutions or countries. This enables research in areas where data are sparse and therefore need to be pooled across different institutions (see next section).

In sum, interoperability can generate new medical insights, making it possible to analyze existing data sources more efficiently. This can advance translational medicine and help to move research discoveries swiftly from the laboratory to the point of care. At a larger scale, it can drive evidence-based practices in medicine and accelerate their implementation into public health policies.

### Interoperability for international cooperation

Interoperable interfaces and standard terminologies make health data exchangeable and comparable across systems, institutions and countries. This has obvious benefits for cross-institutional and international cooperation. As mentioned previously, exchanging health data across different IT systems is especially important when data are scarce or when very large datasets are needed, such as in research on rare diseases, precision medicine or drug development. In the case of rare diseases, for example, the number of patients is often so small that even large health institutions may have access to only a handful of cases of a given disease (sometimes only a single patient). To get a better understanding of these diseases and improve diagnosis and treatment, data exchange across institutions is therefore crucial. National and international networks like the Undiagnosed Diseases Network (UDN)^31^ in the US or the European Reference Networks (ERNs)^32^ already aim to improve collaboration between clinicians treating rare diseases. The adoption of standard data formats and terminologies such as the Orphanet rare disease nomenclature^33^ or the previously mentioned HPO for phenotypic abnormalities^25^ can help to coordinate international efforts to optimize research and care in this area.

Exchanging health data internationally is also essential for tackling global health issues more effectively. Arguably the most serious public health risk – or, for that matter, the most serious risk for humanity in general – is a global pandemic.^34^ Last year’s centenary of the 1918 influenza epidemic should remind us of the potentially devastating consequences of such pandemics, with levels of human suffering and death only comparable to the deadliest wars (the 1918 influenza epidemic, or “Spanish flu”, has been estimated to have killed at least 50 million people worldwide).^35^ In today’s highly connected world, a dangerous epidemic could spread within hours from almost anywhere in the world to Berlin, Beijing or Buenos Aires, killing tens of millions of people within a few months.^36^ But the same global connectedness by which epidemics can spread rapidly around the globe can also help us to control them. If health data are interoperable so that information can be easily exchanged across borders and organizations, effective surveillance systems can be established that allow for an accurate tracking of global disease movements. Outbreaks can then be detected early and further spread prevented.

Importantly, interoperable health IT systems not only facilitate the exchange of data but also the exchange of algorithms, applications and technologies. If organizations standardize their data, cutting-edge health applications developed for these standardized data could be made available to patients and physicians worldwide. The wide use of smartphones and mobile apps can further contribute to the dissemination of digital health technology. This can aid the “democratization” of medicine, making health technologies globally accessible and improving healthcare in underprivileged regions of the world.^37,38^

2019 has seen the first exchange of common data models between European countries (an electronic prescription and a digital patient summary).^39,40^ This demonstrates that cross-border exchange of interoperable health data is attainable, even though it is still a long way to a global infrastructure for interoperable data exchange. But gradually expanding the reach of these and other, more extended data models will eventually advance the international exchange of health data to provide seamless, cross-border care to patients.

## Conclusion and outlook

Digital medicine depends on interoperable and standardized data. In this article, we discussed how interoperable health data can help to realize the full potential of AI and big data, improve the communication of medical information, make medical research more efficient and foster international cooperation. As interoperability requires the collaborative efforts of healthcare professionals, researchers, IT experts, data engineers, and politicians, it is important to make interoperability a prominent topic in medicine and healthcare. Eventually, efforts to improve interoperability will pay huge dividends: With international standards and medical terminologies, interoperability can pave the way for an interconnected digital health infrastructure that overcomes barriers between individuals, organizations and countries. This will make it possible to turn digital medical data into meaningful information and improve the health and well-being of patients worldwide.
